# 

**Published:** 1882-01

**Authors:** 


					﻿voi. xvm.
Januarv 1882.
No. 4.
ThE
IMIIBli
Thad S Up de Graff M.D
e; p t q r ;
ELMIRA.N.Y.
				

